# A Resource-Efficient Multi-Entropy Fusion Method and Its Application for EEG-Based Emotion Recognition

**DOI:** 10.3390/e27010096

**Published:** 2025-01-20

**Authors:** Jiawen Li, Guanyuan Feng, Chen Ling, Ximing Ren, Xin Liu, Shuang Zhang, Leijun Wang, Yanmei Chen, Xianxian Zeng, Rongjun Chen

**Affiliations:** 1 School of Computer Science, Guangdong Polytechnic Normal University, Guangzhou 510665, China; lijiawen@gpnu.edu.cn (J.L.); fengguanyuan@gpnu.edu.cn (G.F.); lingchen@gpnu.edu.cn (C.L.); renximing@gpnu.edu.cn (X.R.); wangleijun@gpnu.edu.cn (L.W.); zengxianxian@gpnu.edu.cn (X.Z.); 2 Hainan Provincial Key Laboratory of Sports and Health Promotion, Hainan Medical University, Haikou 571199, China; 3 Key Laboratory of Emergency and Trauma of Ministry of Education, The First Affiliated Hospital of Hainan Medical University, Haikou 571199, China; 4 Department of Electrical and Computer Engineering, University of Macau, Macau 999078, China; yb87445@um.edu.mo; 5 School of Life Science and Technology, University of Electronic Science and Technology of China, Chengdu 610056, China; zhang.s@njtc.edu.cn; 6 School of Artificial Intelligence, Neijiang Normal University, Neijiang 641004, China; 7 Guangdong Provincial Key Laboratory of Big Data Computing, The Chinese University of Hong Kong, Shenzhen (CUHK-Shenzhen), Shenzhen 518172, China; 8 Guangdong Provincial Key Laboratory of Intellectual Property and Big Data, Guangdong Polytechnic Normal University, Guangzhou 510665, China

**Keywords:** electroencephalography (EEG), multi-entropy fusion, brain rhythms, single-channel, emotion recognition

## Abstract

Emotion recognition is an advanced technology for understanding human behavior and psychological states, with extensive applications for mental health monitoring, human–computer interaction, and affective computing. Based on electroencephalography (EEG), the biomedical signals naturally generated by the brain, this work proposes a resource-efficient multi-entropy fusion method for classifying emotional states. First, Discrete Wavelet Transform (DWT) is applied to extract five brain rhythms, i.e., delta, theta, alpha, beta, and gamma, from EEG signals, followed by the acquisition of multi-entropy features, including Spectral Entropy (PSDE), Singular Spectrum Entropy (SSE), Sample Entropy (SE), Fuzzy Entropy (FE), Approximation Entropy (AE), and Permutation Entropy (PE). Then, such entropies are fused into a matrix to represent complex and dynamic characteristics of EEG, denoted as the Brain Rhythm Entropy Matrix (BREM). Next, Dynamic Time Warping (DTW), Mutual Information (MI), the Spearman Correlation Coefficient (SCC), and the Jaccard Similarity Coefficient (JSC) are applied to measure the similarity between the unknown testing BREM data and positive/negative emotional samples for classification. Experiments were conducted using the DEAP dataset, aiming to find a suitable scheme regarding similarity measures, time windows, and input numbers of channel data. The results reveal that DTW yields the best performance in similarity measures with a 5 s window. In addition, the single-channel input mode outperforms the single-region mode. The proposed method achieves 84.62% and 82.48% accuracy in arousal and valence classification tasks, respectively, indicating its effectiveness in reducing data dimensionality and computational complexity while maintaining an accuracy of over 80%. Such performances are remarkable when considering limited data resources as a concern, which opens possibilities for an innovative entropy fusion method that can help to design portable EEG-based emotion-aware devices for daily usage.

## 1. Introduction

Emotions are fundamental components of human psychological processes that shape thinking patterns, behavior, and decision making and play a critical role in social interaction, learning, and work. As humans increasingly rely on intelligent systems, the advancement of emotion recognition technologies is poised to revolutionize human–computer interaction. Thus, integrating emotional intelligence into such systems can significantly improve the efficiency of human–machine communication [1]. Furthermore, gaining a deeper understanding of the intrinsic mechanisms of emotions is beneficial for mental health, as it facilitates the development of accurate emotion monitoring methods while paving new pathways for the prevention and treatment of mental disorders such as depression and anxiety [2]. While emotions can be detected through external cues like facial expressions, speech, and other body language, social norms, personal contexts, and habits often obscure actual expressions, which are current challenges for precise emotion recognition [3].

Usually, a model capable of quantifying emotions is first needed to achieve emotion recognition. On the one hand, a discrete model divides emotions into a series of distinct emotional entities, such as happiness, contentment, sadness, and anger. Nonetheless, there still needs to be an academic consensus on the exact number of these basic emotions [4]. In addition, discrete emotion models struggle to describe continuous changes in emotions. On the other hand, a continuous model defines emotions through continuous dimensions, such as arousal and valence, better representing the variations in emotions. Specifically, valence refers to the pleasantness of emotion, ranging from negative (e.g., sadness or anger) to positive (e.g., happiness or contentment), and arousal denotes the level of emotional excitement, ranging from low (e.g., calmness or relaxation) to high (e.g., excitement or tension) [5]. Second, less masked inputs like physiological signals are preferred to improve emotion recognition accuracy. For instance, electroencephalography (EEG), electrooculography (EOG), and electrocardiography (ECG) signals are naturally generated by the body’s systems. They directly reflect emotional reactions and are less easily influenced by human factors [6]. Typically, EEG signals, which record electrophysiological signals of neuronal activity in the brain, objectively contain response behaviors [7]. They provide a clear description of emotional expression, as their high-temporal-resolution record changes at the millisecond level, which makes them appropriate for detecting instantaneous variations in response to emotions triggered by specific stimuli or situations, demonstrating their portability and cost-effectiveness for the design of portable devices in emotion recognition [8].

As for EEG-based emotion recognition, data pre-processing, feature extraction, and classification are vital steps, and performance evaluation is essential for methodological validation. Initially, data pre-processing is necessary, as EEG signals are highly susceptible to noise and interference from other physiological signals. To this end, Independent Component Analysis (ICA) can denoise and remove artifacts [9]. In addition, raw EEG signals are segmented into several slices of varying lengths to include short-term fluctuations that sense emotional changes [10]. Therefore, pre-processed EEG signals provide a foundation for subsequent feature extraction, which aims to quantify and express the overt and latent information in the EEG signals. In this regard, these features are typically divided into three categories: time-domain (e.g., mean, standard deviation, and first-order difference [11]), frequency-domain (e.g., power spectral density [12]), and nonlinear (e.g., asymmetry, autocorrelation, zero-crossing rate, and entropy [13]) features. In light of the intricate and multifaceted nature of EEG signals, integrating different features to encapsulate their inherent characteristics is often imperative [14]. Moreover, the advance of deep learning has enabled the development of automated feature extractors based on Convolutional Neural Networks (CNNs) and Long Short-term Memory (LSTM) [15]. Classification aims to categorize emotional states based on extracted features. Generally, classifiers can be divided into two categories: conventional machine learning classifiers and deep learning models. Among conventional classifiers, Support Vector Machine (SVM), Random Forest (RF), k-nearest Neighbors (kNN) [16], and XGBoost [17,18,19] are widely utilized. For instance, SVM is particularly effective in addressing nonlinear problems, especially when dealing with limited datasets. RF enhances classification stability by combining the outputs of multiple decision trees. kNN is a nonparametric algorithm that makes no assumptions about the underlying data distribution that benefits biomedical signals like EEG, as it exhibits complex, nonlinear patterns across different emotions. XGBoost, a gradient-boosting framework, has demonstrated strong performance in EEG-based emotion recognition tasks by leveraging its efficiency and ability to handle imbalanced data. On the other side, deep learning models such as CNN, LSTM, Temporal Convolutional Network (TCN), and Graph Convolutional Neural Network (GCNN), are well-suited for handling high-dimensional data [20]. While deep learning models typically outperform conventional classifiers on large datasets, they usually require more computational costs and training time. Finally, the performance of the features and classifiers is assessed in a comparative study, where the most commonly used metrics include accuracy, precision, recall, F1 score, and the Receiver Operating Characteristic (ROC) curve [21]. Thus, a comprehensive evaluation ensures that the classifier or model demonstrates adequate generalization and stability for emotion recognition.

Recent studies have been reported based on the above steps. For example, Trujillo et al. [11] extracted 1086 time-domain and frequency-domain features from EEG signals and assessed them through feature selection combined with various classifiers. They claimed that the RF classifier paired with Kernel Principal Component Analysis (KPCA) offered an accuracy of 93.20%. Yu et al. [12] downsampled, filtered, and designed a short time window to analyze the temporal dependence in EEG signals by combining an attentional mechanism with LSTM, which provided accuracies of 85.40% and 74.26% on the SEED dataset and SEED-IV datasets, respectively. Zong et al. [19] constructed a hybrid FCAN-XGBoost model for EEG-based emotion recognition. It achieved 95.26% and 94.05% accuracy on DEAP and DREAMER, respectively. Fernandes et al. [22] investigated time-domain and frequency-domain features, employing deep learning models and conventional machine learning classifiers. They found that combining differential entropy with Dynamic Graph Convolutional Neural Networks (DGCNNs) yielded an accuracy of 89.97% across various frequencies. In comparison, kNN only achieved an accuracy of 73.23%, indicating the advantages of deep learning models in optimizing classification performance, although they necessitated more prolonged processing procedures. Song et al. [23] proposed an EEG-based Variational Instance-Adaptive Graph (V-IAG) method to address individual differences and uncertainty in the dynamic relationship between brain regions. By extracting the energy features of each EEG channel and constructing a fractional variational-instance adaptive graph combined with a multi-layer multi-graph neural network model, they obtained accuracies of 92.82% and 93.09% in the classification of valence and arousal, respectively. In addition, they stated that emotions exhibited a complex dynamic relationship with individualized modeling and brain regions and that integrating this relationship with graph neural networks can effectively improve the accuracy of emotion recognition. García-Hernández et al. [24] applied a genetic algorithm to select an optimal subset from the 2548 features, then compared the performances utilizing kNN, RF, and an Artificial Neural Network (ANN), which achieved accuracies of 90.06%, 93.62%, and 95.87%, respectively. Such results reveal that feature optimization and methodological comparison can beneficially identify the best approaches for EEG-based emotion recognition. Finally, Padhmashree and Bhattacharyya [25] adopted Multivariate Variational Mode Decomposition (MVMD) to extract modulated oscillations from multi-channel EEG signals, where the time-frequency images were generated by combining Joint Instantaneous Amplitude (JIA) and Joint Instantaneous Frequency (JIF). These time-frequency images were fed into a Residual Network (ResNet)-18 model to extract valuable features, followed by a softmax layer for emotion recognition. This framework offered classification accuracies of 99.03% for arousal and 97.75% for valence, demonstrating its ability to extract complex EEG features for emotion recognition.

**Figure 1:**
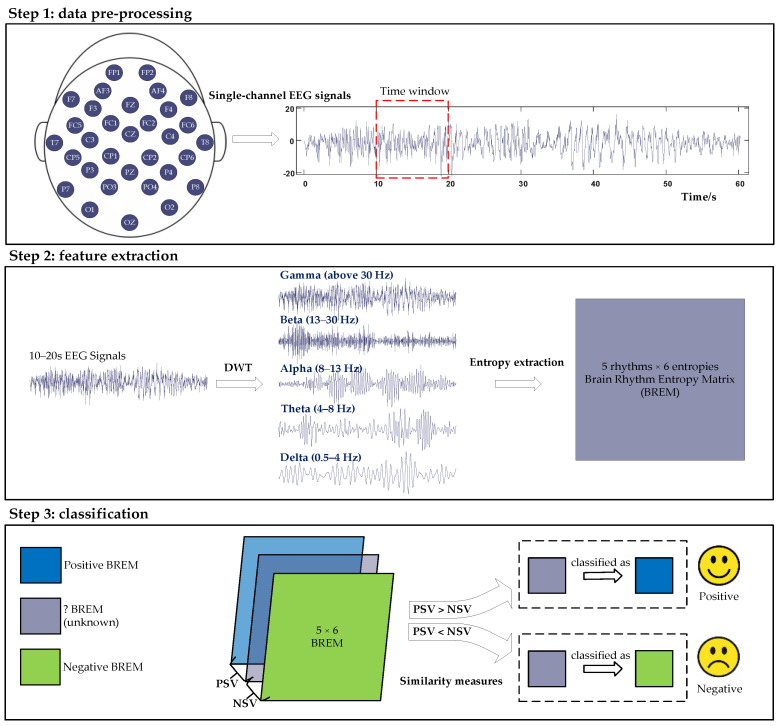
The overall framework of the proposed resource-efficient multi-entropy fusion method for EEG-based emotion recognition.

The above studies reported various techniques for EEG-based emotion recognition, each with strengths, but they also presented drawbacks, especially in the case of a data resource-efficient classification. Many deep learning models, such as DGCNN and ResNet-18, demand substantial computational resources, limiting their deployment in resource-constrained environments. In addition, their complexity may lead to overfitting, particularly with smaller datasets, and the required extensive hyperparameter tuning can make them less practical compared to more straightforward solutions. Moreover, several studies could have improved the interpretability of advanced models, complicating the understanding of their decision-making processes. In this regard, multi-entropy fusion yields more interpretable results, as the entropy feature originates from information theory and mainly represents the uncertainty or complexity of an EEG system. It is also less sensitive to artifacts and noise, allowing it to present small fluctuations in brain activity, which are beneficial for analyzing emotional states and enhancing practical applications in real-world scenarios. However, prior works have yet to fully consider the effective achievement of multi-entropy fusion under conditions of limited data resources. To this end, this work proposes a resource-efficient multi-entropy fusion method for classifying emotional states, the novelty of which is the similarity measures of the Brain Rhythm Entropy Matrix (BREM), which involves multi-entropy feature extraction and similarity measure-based classification to improve the accuracy and interpretability of emotion recognition. For a better illustration, the overall framework is depicted in Figure 1.

The first step is data pre-processing, which comprises two operations: filtering and slicing. Filtering eliminates potential interference from ECG and EOG signals [26]. Then, to further optimize the data size and adapt it for portable devices, this work thoroughly investigates a series of segments with varying time lengths (5 s, 10 s, 20 s, and 30 s) for the identification of a suitable time window in the proposed method.

The second step is feature extraction, which contains three phases: obtaining brain rhythms by Discrete Wavelet Transform (DWT), extracting multi-entropy features, and producing the BREM through data fusion. As for emotion processing, negative emotions are primarily processed in the subcortical nuclei, temporal lobe, temporoparietal junction area, and inferior frontal gyrus [27]. Similarly, hyperactivity in the left prefrontal lobe is generally associated with positive emotions, while hyperactivity in the right prefrontal lobe is linked to negative emotions [28]. Such behaviors suggest that the forebrain plays a pivotal role in emotion recognition. In addition, there is a strong correlation between emotional states and brain rhythms: delta (0.5–4 Hz), theta (4–8 Hz), alpha (8–13 Hz), beta (13–30 Hz), and gamma (above 30 Hz) [29]. For instance, delta power increases when the subject is in a highly relaxed or emotionally depressed state [30]. Although time-domain and frequency-domain features provide deep insights into emotional changes by analyzing brain rhythms and brain regions, they are insufficient for indicating the subtle complexity of emotional shifts [31]. Therefore, the proposed method adopts entropy as a quantitative characteristic that extracts six types—Spectral Entropy (PSDE), Singular Spectrum Entropy (SSE), Sample Entropy (SE), Fuzzy Entropy (FE), Approximation Entropy (AE), and Permutation Entropy (PE)—from five brain rhythms and fuses them into a 5 × 6 matrix on a single channel, denoted as the BREM, which offers highly interpretable representations of emotional variations by considering the rhythmic characteristics in the entropy scale.

The third step is establishing a specialized classification method by applying the similarity measures of the BREM between the unknown testing data and positive/negative emotional samples. Specifically, a higher similarity indicates more significant information overlap, implying they are in the same category, while a lower similarity reveals they are less likely to be the same [32,33,34]. Since BREM includes the details regarding brain rhythms and entropies, similar BREMs show similar emotional states, providing an interpretable basis for EEG-based emotion recognition through the multi-entropy view. In addition, unlike other classification methods, such an approach does not rely on large amounts of data for training. Instead, it requires only positive and negative samples for similarity measures, making it more resource-efficient. Note that there are different methods for similarity measures, so selecting the appropriate one is vital for the classification task.

Finally, in the evaluation step, this work assesses the single-channel mode and the single-region mode for emotion recognition. The single-region mode is created by fusing the BREMs from the channels located in the same brain region, and the single-channel mode applies the BREM to one specific channel only. By evaluating the data inputs from a single channel or single region, the results provide an in-depth understanding of how the BREM can be effectively improved for emotion recognition through the use of a few data resources to establish a resource-efficient multi-entropy fusion method. The contributions of this work are summarized as follows:

1. We propose the use of highly interpretable BREM data through a multi-entropy fusion approach to represent emotional EEG signals and the employment of a similarity-based classification approach to reduce the required sample size and the complexity of the training process.
2. We identify a suitable similarity measure method for classifying the BREMs from various emotional states, enhancing the performance of the proposed method.
3. We investigate the most suitable length (5 s, 10 s, 20 s, or 30 s) and the most resource-efficient data input mode (single-channel or single-region mode) to minimize data sources while maintaining an accuracy of over 80%.

The rest of this work is organized as follows: Section 2 describes the DEAP dataset and the valence–arousal emotional model. Subsequently, the details of the BREMs derived from multi-entropy fusion and the resource-efficient classification method through similarity measures are presented in Section 3. Section 4 shows the results and discussion, including statistical analysis, classification results, appropriate time-segment and channel results, a comparative study, and discussion. Finally, the conclusion and future work are contained in Section 5.

## 2. Experimental Dataset

The experimental data are from the DEAP dataset [35]. It contains EEG signals and corresponding emotion labels. The key information regarding the DEAP dataset is listed in Table 1. In total, 32 subjects (15 females and 17 males) participated in the experiment, with a mean age of 27.19 ± 4.45 years. Subjects were asked to watch 40 one-minute music videos with a Self-Assessment Manikin (SAM) to rate each music video according to their valence and arousal levels on a scale of 1–9. Moreover, different emotional states can be mapped into a coordinate system to form an emotional space consisting of valence and arousal, i.e., a valence–arousal model, where a valence score ≥ 5 indicates High Valence (HV) a score < 5 indicates Low Valence (LV). High Arousal (HA) and Low Arousal (LA) are similarly categorized based on score of 5 [36]. While watching music videos, EEG signals were recorded using a 32-channel system under a sampling frequency of 128 Hz. It is worth noting that EEG signals provided by the DEAP dataset were pre-processed with careful attention to minimizing noise, enabling their usage without artifact removal [37,38,39].

**Figure 2:**
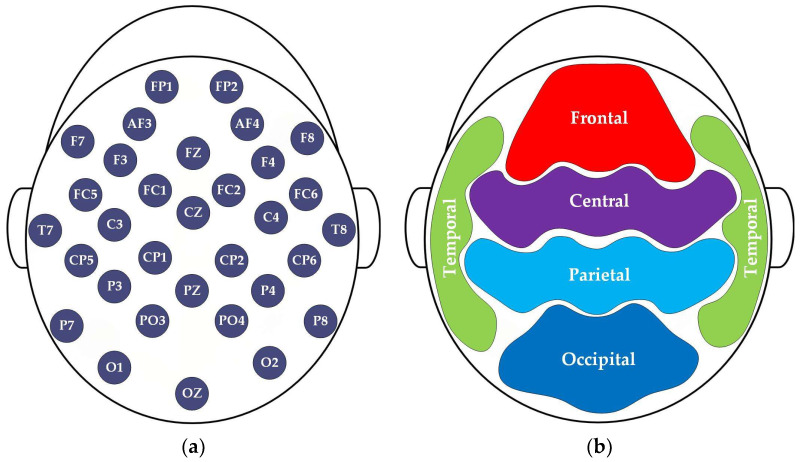
Channel and region locations in DEAP: (**a**) 32 EEG channels; (**b**) five brain regions.

In addition, a balanced distributed sample is beneficial for methodological validation. The DEAP dataset exhibits a ratio of HA/HV (positive) to LA/LV (negative) approaching 1:1, reducing the risk of overfitting. Meanwhile, in order to investigate the resource-efficient data input mode based on a single channel or single region, the 32-channel system is categorized either by electrode positions, as shown in Figure 2a, or by brain regions in terms of anatomical distribution, as illustrated in Figure 2b. Here, each brain region is represented by a set of channels located within that region, with specific channels outlined in Table 2. Due to the low spatial resolution of electrodes, signals from a small surrounding area near the channels are also included in the regional representation.

## 3. Proposed Method

### 3.1. Feature Extraction

First, DWT is adopted to extract the brain rhythms from EEG. Unlike the commonly used transform approaches in signal processing, such as the Short-Time Fourier Transform (STFT), DWT offers multi-resolution analysis through time-frequency decomposition. This property allows DWT to adaptively focus on both high-frequency details and low-frequency trends in the signal, making it well-suited for the processing of EEG and sufficiently able to obtain local features at different time scales [40]. Furthermore, DWT’s hierarchical decomposition enables it to preserve both time-domain and frequency-domain information, which is critical for analyzing the non-stationary nature of EEG. As a result, five brain rhythms, which correlate with diverse emotional states, are extracted. Specifically, DWT involves convolving the signal with a set of wavelet basis functions and extracting the rhythmic features from different frequency bands through multi-layer decomposition and reconstruction, as presented in (1):

(1) $$X(j,k)=\sum_{n}x[n]2^{j/2}\psi [2^{j}n-k]$$

where *X*(*j*, *k*) denotes the wavelet coefficients, which consist of the low-frequency approximation coefficient (*A_i_*) and the high-frequency detail coefficient (*D_i_*); *j* represents the scale level; *k* is the displacement on scale *j*; and *x*[*n*] represents the input EEG signals. Meanwhile, *ψ* represents the discretized wavelet basis function used in the analysis.

**Figure 3:**
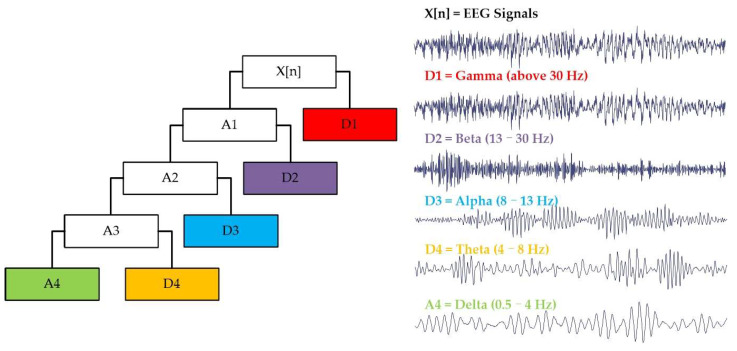
The 4-level DWT extracts five brain rhythms from emotional EEG signals.

In this work, the Daubechies 4 (db4) wavelet is employed as the basis function for DWT due to its ability to balance time and frequency resolution well. Moreover, the compact support and symmetry of the db4 wavelet allow it to efficiently capture the non-stationary nature of EEG, making it well-suited for emotion recognition. An EEG signal from the DEAP dataset is applied to illustrate the DWT in the proposed method, with a sampling frequency of 128 Hz. Technically, emotional EEG signals can be decomposed and reconstructed through a four-level DWT to extract the five brain rhythms, as displayed in Figure 3.

Subsequently, entropy is viewed as a key, since it possesses the uncertainty, complexity, and regularity of EEG signals [41]. Mainly, with respect to multi-entropy fusion, PSDE, SSE, SE, FE, AE, and PE are extracted to indicate the dynamic characteristics of five brain rhythms comprehensively. Mathematically, their calculations are expressed as follows:

*PSDE* quantifies the uncertainty in the frequency distribution of a signal and helps analyze the complexity of rhythmic activities under various emotions [42]. It is derived from the Power Spectral Density (PSD), measuring how signal energy is distributed across different frequency components, as presented in (2):

(2) $$PSDE=-\sum_{i=1}^{N}p(f_{i})\log(p+\varepsilon )$$

where *p*(*f_i_*) is the normalized PSD that uses Welch’s method for energy metrics and *ε* is a value employed to avoid the *log*(0) case.

*SSE* is based on a signal’s Singular Value Decomposition (SVD). It mainly assesses the structural complexity of the time series rather than just its frequency content by examining the singular values obtained from the decomposition; in particular, it shows high sensitivity in processing brain activities during emotional states [43], as presented in (3):

(3) $$SSE=\sum_{i=1}^{r}\tilde{\sigma_{i}}log(\tilde{\sigma_{i}}+\varepsilon )$$

where $\tilde{\sigma_{i}}$ is the normalized singular value. If the singular value distribution is uniform, the signal is complex and has a larger value. On the contrary, if the signal is regular, it has a small value.

*SE* reflects the complexity and regularity of time-series signals by determining the likelihood that similar patterns will remain similar when the data are measured at different time points. It does not require data to be stationary, making it particularly suitable for physiological signals assessing complexity, like EEG [44]. In addition, it is sensitive to noise and outliers, as presented in (4):

(4) $$SE(m,r)=-ln(\frac{\phi (m,r)}{\phi (m+1,r)})$$

where *m* refers to the embedding dimension, *r* is the control threshold, and *ϕ* denotes the number of statistically similar vector pairs.

*FE* extends the concept of SE by incorporating fuzziness into the measure. It evaluates the uncertainty of the signal’s state based on the degree of membership of patterns to sets. Thus, it not only captures subtle differences in patterns due to its fuzzy nature but also shows robustness to noise and variability in data [45], as presented in (5):

(5) $$FE(m,n,r)={lim}_{N\to \infty }[\phi^{m}(n,r)-\phi^{m+1}(n,r)]$$

where *n* represents the fuzzy weights.

*AE* assesses the randomness and variation patterns of time-series data by comparing sequences of points to detect patterns. It is sensitive to the choice of parameters such as the embedding dimension and tolerance [46], as shown in (6):

(6) $$AE(m,r)={lim}_{N\to \infty }[\phi^{m}(r)-\phi^{m+1}(r)]$$

Lastly, *PE* evaluates the complexity of time series by analyzing the order of values rather than their actual magnitudes. It considers the patterns formed by the ranks of the data points and is robust to noise, so it effectively analyzes EEG signals that exhibit chaotic properties [47], as shown in (7):

(7) $$PE\left(x\right)=-\sum_{j=1}^{k}P_{j}\ln\left(P_{j}\right)$$

where *P_j_* is the probability of occurrence of the *j*-th alignment pattern; meanwhile, the range of values of *PE* is [0, ln(*N_p_*)], with smaller values indicating a more structured signal and larger values referring to a more disordered signal.

### 3.2. Multi-Entropy Fusion

By integrating six extracted entropies, multi-entropy fusion offers a comprehensive understanding of changes in brain rhythms, particularly in the context of EEG-based emotion recognition. This approach leverages the strengths of each measure: SSE and PSDE complement each other by revealing the dynamic structure of EEG signals, capturing how brain activity evolves in the time scale; FE and AE delve deeper into the complexity and uncertainty of these signals, offering insights into the regularity and predictability of emotional responses; PE excels at identifying nonlinear features in EEG, which are often crucial for detecting subtle emotional states; and SE quantifies energy distribution across different frequency bands, showing a broad view of frequency-domain characteristics that are essential for assessing the stability and energy patterns of brain rhythms. This nuanced fusion not only enhances the understanding of EEG characteristics associated with emotional changes but also helps to identify specific brainwave patterns that correlate with distinct emotional states, making it a powerful solution in affective neuroscience. Thus, the proposed method incorporates feature-level fusion to generate the BREM.

Another aim is to investigate the single-channel mode and single-region mode for emotion recognition, resulting in variations in BREM generation for each mode. In the single-channel mode, BREM contains 30 features, representing a combination of five brain rhythms and six entropies for a single EEG channel. Therefore, the size in this mode is 5 × 6, as presented in (8). As for the single-region mode, the BREM is formed by fusing the channels within the same brain region. For example, regarding the occipital region, the BREMs from PO3, PO4, O1, O2, and OZ are combined, resulting in a larger matrix size of 5 × 30, as shown in (9). Following this approach, the BREMs of five brain regions are generated by fusing the corresponding EEG channels listed in Table 2, providing a comprehensive feature representation. This fusion strategy facilitates in-depth analysis of the channel number in emotion recognition by involving various BREMs, which helps to determine the appropriate data size for accomplishing a resource-efficient method.

(8) $${BREM}_{single-channel}=\left[\begin{array}{cccccc} \gamma_{AE} & \gamma_{FE} & \gamma_{SE} & \gamma_{PE} & \gamma_{SSE} & \gamma_{PSDE}\\ \beta_{AE} & \beta_{FE} & \beta_{SE} & \beta_{PE} & \beta_{SSE} & \beta_{PSDE}\\ \alpha_{AE} & \alpha_{FE} & \alpha_{SE} & \alpha_{PE} & \alpha_{SSE} & \alpha_{PSDE}\\ \theta_{AE} & \theta_{FE} & \theta_{SE} & \theta_{PE} & \theta_{SSE} & \theta_{PSDE}\\ \delta_{AE} & \delta_{FE} & \delta_{SE} & \delta_{PE} & \delta_{SSE} & \delta_{PSDE} \end{array}\right]$$

(9) $${BREM}_{occipitalregion}=\left[\begin{array}{ccc} {BREM}_{PO3} & {BREM}_{PO4} & \begin{array}{ccc} {BREM}_{O1} & {BREM}_{O2} & {BREM}_{OZ} \end{array} \end{array}\right]$$

where *γ*, *β*, *α*, *θ,* and *δ* denote gamma, beta, alpha, theta, and delta, respectively.

### 3.3. Classification Method

Concerning biological sequences, the degree of similarity is a fundamental measure of the extent of resemblance between them. Inspired by this concept, this work puts forth a classification method based on the similarity measures of the BREM. For instance, unknown BREM data are compared with a positive BREM sample (HA/HV) and a negative sample (LA/LV) to determine similarity measures between them. If the unknown BREM exhibits a greater degree of similarity with the positive sample, this indicates that the testing data can be characterized as HA/HV. Then, four commonly used methods in this field, namely Dynamic Time Warping (DTW), Mutual Information (MI), the Spearman Correlation Coefficient (SCC), and the Jaccard Similarity Coefficient (JSC), are chosen to investigate in this work. The details are outlined as follows:

*DTW* is a nonlinear matching algorithm for comparing time-series signals that can perform dynamic alignment on the time axis to minimize the distance between two data points [48]. It mainly focuses on the shape of the time-series signals rather than their absolute values, which beneficially handle data of different lengths and temporal distortions, as shown in (10) and (11):

(10) $$C(i,j)=d(A_{i},B_{j})+min\{C(i-1,j),C(i,j-1),C(i-1,j-1)\}$$

(11) DTW(A,B)=C(n,m)

where *A* and *B* are two data point that are needed to calculate the similarity lengths of *n* and *m*; *d* is the local distance between *A_i_* and *B_j_* at points *i* and *j*, respectively; *C* is the cumulative distance matrix; and *C*(*n*, *m*) is denoted as the value of the last element in the *C* matrix. The smaller the *DTW*, the higher the similarity between the BREMs.

*MI* measures the dependency between two data points, and it can demonstrate how much information they share [49]. Thus, by comparing the BREMs of different emotional states, the trend of entropy change can be indicated, as shown in (12):

(12) MI(A;B)=H(A)+H(B)−H(A,B)

where *H* denotes the entropy of data points *A* and *B* and *H*(*A*, *B*) represents the joint entropy between them.

*SCC* is a nonparametric measure of rank correlation that assesses how well a monotonic function can describe the relationship between two variables. It helpfully evaluates the degree to which the relationship between two variables can be expressed as a linear relationship after ranking the data [50], as shown in (13):

(13) $$SCC=1-\frac{6\sum_{i=1}^{n}{\left(A_{i}-B_{i}\right)}^{2}}{n(n^{2}-1)}$$

Lastly, *JSC* is a measure of the similarity between two sets. It is commonly used to compare finite discrete sets by calculating the ratio of the intersection to the union of the sets, effectively identifying how much the two sets overlap [51]; its values range from 0 (no similarity) to 1 (entire similarity), as shown in (14):

(14) $$JSC=1-\frac{\left|A\cap B\right|}{\left|A\cup B\right|}$$

To achieve a resource-efficient classification method using similarity, a template that serves as either a positive or negative sample is essential, functioning as a benchmark. Meanwhile, after thoroughly considering inter-individual variability and the differences in emotions elicited by watching music videos in the DEAP dataset, the Leave-One-Out Cross-Validation (LOOCV) approach is employed to choose the templates. For instance, for subject S1, out of 40 trials, 16 of them are LA, and 24 are HA. Thus, the BREMs from the first LA and the first HA trials are selected as positive and negative templates, respectively. In contrast, the remaining trials are applied as unknown testing data in the similarity measures to classify their emotional state as LA or HA. Next, the BREMs of the second LA and the first HA trials are selected as templates, followed by sequential cyclic analysis. Based on that, 16 × 24 evaluations are conducted, employing similarity measures per subject. In addition, as for evaluation metrics, the accuracy is defined as follows:

(15) $$Accuracy=\frac{TN+TP}{TN+FN+TP+FP}$$

where *TP* refers to the correctly predicted positive examples, i.e., instances classified as HA or HV. *FP* represents the incorrectly predicted positive examples, i.e., HA or HV instances misclassified as LA or LV. *FN* denotes the incorrectly predicted negative examples, i.e., LA or LV instances misclassified as HA or HV. Finally, *TN* represents correctly predicted negative examples, i.e., instances correctly classified as LA or LV.

## 4. Results and Discussion

In this work, the experimental results of the proposed method are programmed by MATLAB. To facilitate reproducible research and positively affect the academic field, the codes are freely available at https://github.com/zyzc75/BREM-SIMILARITY.git (accessed on 18 January 2025).

### 4.1. Statistical Analysis

**Figure 4:**
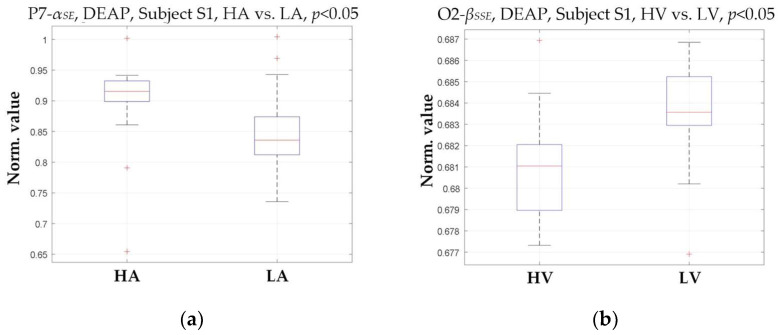
Two examples of ANOVA test box plots for the best entropy features that provide the highest accuracy from subject S1 in the DEAP dataset. (**a**) The alpha sample entropy (*α_SE_*) in the P7 channel for arousal classification; (**b**) the beta singular-spectrum entropy (*β_SSE_*) in the O2 channel for valence classification.

First, a qualitative analysis is performed to demonstrate the statistical significance of the selected entropy features in relation to emotional states. Specifically, Analysis of Variance (ANOVA) is a commonly used statistical verification method that evaluates differences in group means and variances. When the resulting *p*-value is equal to or smaller than the significance level (typically 0.05), the feature exhibits a significant difference concerning specific emotional variations. It is, therefore, suitable for classification tasks. Figure 4 presents two examples of ANOVA test box plots for the best entropy features that provide the highest accuracy from subject S1 in the DEAP dataset. In Figure 4a, the alpha-sample entropy (*α_SE_*) in the P7 channel is selected for arousal classification. In contrast, in Figure 4b, the beta-singular spectrum entropy (*β_SSE_*) in the O2 channel is chosen for valence classification. Their *p*-values are less than 0.05, so the statistical analysis indicates significant differences in the emotional categories.

In Figure 4, higher values of P7-*α_SE_* predominantly appear in the HA state for arousal classification. In comparison, lower values are found in the LA state, suggesting a positive correlation between arousal level and the presence of P7-*α_SE_* in the BREM for subject S1. Similarly, the appearance of O2-*β_SSE_* in the BREM for valence classification provides informative clues indicating valence levels. In this work, the BREM is not subjected to feature selection to retain the comprehensiveness of the entropy features while ensuring classification accuracy. Nonetheless, several features may not exhibit statistical significance for emotion recognition. Consequently, this statistical analysis only focuses on those entropy features selected based on specific channels with the highest accuracy rather than all extracted features.

Following this approach, ANOVA tests are conducted on the entropy features selected from each channel for all 32 subjects meeting the same conditions in the DEAP dataset. The overall results indicate that their *p*-values are all less than 0.05, showing trends similar to those of the two examples illustrated in Figure 4, demonstrating statistical significance. Such statistical results confirm the rationale for selecting valuable entropy features and provide optimal support for robust classification.

### 4.2. Classification Results

The mean ± standard deviation (%) of accuracy for arousal and valence classification employing different time windows, similarity measures, and data input modes are summarized in Table 3. The best results are underlined in bold.

As shown in Table 3, SCC and JSC are less effective than the other methods, since the average accuracy of these two methods is below 72% and the standard deviation is around 10%. For example, JSC remains at 59.38 ± 16.26% and 56.91 ± 9.70% for 30 s time lengths for arousal and valence classification, respectively. The analysis demonstrates that, taking the arousal classification of subject S1 with 40 trials (16 LA and 24 HA) as an example, 15 LA and 23 HA samples need to be tested after selecting positive and negative samples. Employing JSC for similarity measures, all testing samples are misclassified as HA, resulting in a fixed classification accuracy of 23/(15 + 23) = 60.53%. This pattern is consistent across other subjects, so the mean and standard deviation remain fixed, regardless of changes in time windows, leading to performance failure. The failure of the JSC method is likely due to its limitations. JSC is suitable for measuring the degree of overlap between two sets, but the multi-entropy features in EEG signals are dynamic and complex. The changes under different emotional states are not simple set relationships or linear. As a result, it is not good enough to effectively capture such complex dynamic characteristics. In addition, the SCC mainly measures the rank correlation of two BREMs, i.e., their monotonic relationship. Nonetheless, emotional changes are often not monotonic, and EEG signals show nonlinear properties under different emotional states. Therefore, the SCC is improper for obtaining such changes in the BREM and only measures similarity according to the change in rank, making it ineffective at discerning the subtle differences between emotions, resulting in poorer performance.

On the other side, DTW adaptively addresses temporal data deformation. It effectively includes the nonlinear characteristics of change between disparate time segments in EEG signals, demonstrating high accuracy and stability in emotion recognition. MI reflects the relationship between EEG signals by quantifying the information shared between two variables, yielding promising results in emotion recognition. Consequently, nonlinear similarity measure methods, such as DTW and MI, are appropriate in this work. Overall, DTW shows stability in valence classification, as indicated by a smaller standard deviation. Meanwhile, it maintains balanced performance in arousal classification. Therefore, considering its accuracy and stability, DTW can be regarded as a suitable similarity measure method for the BREM to perform resource-efficient classification.

After settling the DTW, it is observed that as the time window progressively decreases, accuracy improves for both valence and arousal, accompanied by a reduction in the standard deviation. In particular, the performance of a 5 s time window is up to 80%, whether in a single channel or single region. To be precise, it improved by nearly 3% compared to a 30 s window and by almost 1% compared to both 10 s and 20 s windows. This improvement may be due to the instantaneous nature of emotional changes, which experience significant fluctuations over a relatively short period. On the other hand, a longer time window may contain more information but include additional irrelevant signals, which may reduce the prominence of these instantaneous fluctuations. As a result, a 5 s window is selected as the stationary segment for emotion recognition, consistent with previous work [52].

Next, the experimental results demonstrate that employing a single-channel input produces better outcomes than single-region input. Specifically, the arousal classification accuracy of the single-channel mode using DTW with a 5 s time window is 1.56% higher than that of the single-region mode. In comparison, the standard deviation is 0.74% lower. Similarly, in valence classification, the accuracy of the single-channel mode is 2.79% higher, with a standard deviation that is 0.05% lower. This enhancement is attributed to the single channel’s ability to directly involve multi-entropy features associated with specific emotional dimensions, which helps to minimize potential interference and redundant information. Conversely, integrating multiple channels introduces complexity that may dilute the contribution of effective signals from individual channels. Although the difference is insignificant, single-channel data input reduces computational cost and model training time, accentuating the practical advantages of single-channel input in the proposed method, which is a primary concern when designing portable devices. Thus, the characteristics of the single-channel mode are the focus of the proposed method.

### 4.3. Appropriate Time-Segment and Channel Results

When selecting DTW as the similarity measure method for single-channel BREMs derived from a length of 5 s as input, the subsequent investigation concentrates on the appropriate time segment and channel for EEG-based emotion recognition. The results of 32 subjects from the DEAP dataset are listed in Table 4, based on which the best combination of each subject can be determined.

**Figure 5:**
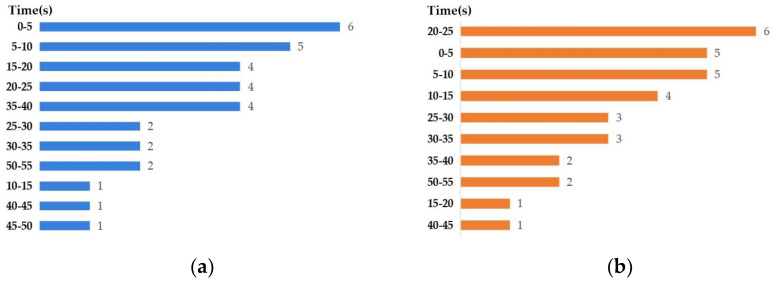
Statistical frequency of the optimal time segment (s) for 32 subjects from the DEAP dataset: (**a**) arousal classification; (**b**) valence classification.

Table 4 reveals that the appropriate time segment and channel for arousal and valence classification vary among the subjects, implying individual differences in EEG responses to emotional stimuli. For arousal classification, the appropriate time segment is predominantly concentrated in the early stage of the experiment (0–20 s for 16 subjects) and the middle stage (20–40 s for 12 subjects), with the highest classification accuracy for S21, reaching 94.74% in 0–5 s. It also follows a similar trend for valence classification, where the highest classification accuracy of 89.47% appears in 20–25 s (S6) and 30–35 s (S23). In addition, Figure 5 depicts the statistical frequency of the optimal time segment for 32 subjects from the DEAP dataset. Such findings as those reported in Table 4 and Figure 5 align with the nature of the two emotional dimensions. Arousal reflects an individual’s immediate response to emotional stimuli, which tends to be most prominent in the initial or middle periods [53]. In contrast, valence is associated with the individual’s overall emotional evaluation and response, which may require more time for processing and assessment by the subject themselves [54].

**Figure 6:**
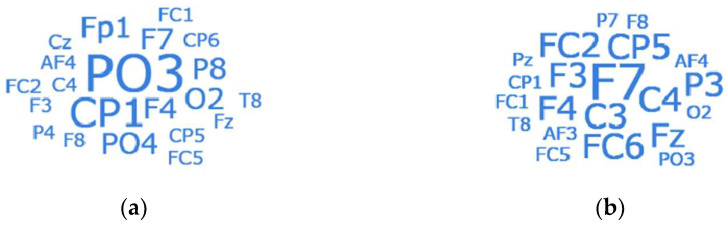
Word clouds of the representative channels for 32 subjects from the DEAP dataset: (**a**) arousal classification; (**b**) valence classification.

For better understanding, Figure 6 illustrates word clouds of the identified appropriate channels, indicating that most are located in the central, occipital, and frontal regions when conducting arousal and valence classifications. On the one hand, such regions are typically associated with sensory and attentional processing, given their role in arousal levels, which aligns with the function of the occipital region (responsible for visual processing), the central region (linked to motor control and sensory integration), and the frontal region (also involved in the cognitive evaluation of emotional stimuli, playing a central role in emotional regulation). Consequently, they facilitate the integration of the brain’s immediate response to stimuli, contributing to effective arousal classification. On the other hand, regarding valence, the channel results exhibit slight differences, with higher occurrences of F7, C3, and C4, which aligns with the conclusion that the frontal region is associated with social behavior, decision making, and emotional control. Specifically, the central part of the brain is part of the motor cortex, which is involved in motor expression and is closely related to emotional expression, including facial expressions and body language. Moreover, the discovered channels are not identical among all subjects, indicating that cognitive processing and the cooperation of multiple brain regions are typically required to assess emotion. For example, the prefrontal cortex modulates activity in brain regions that produce emotions, with downregulation and upregulation enhancing activity in areas associated with emotional experiences, such as the amygdala and the prefrontal lobes [55]. The prefrontal lobe’s location corresponds with that of F7, C3, and C4, situated on the left side of the frontal lobe and extending forward, implying a close relationship with the activity associated with downregulation and upregulation in emotions.

Based on the above observations, it is plausible to speculate that arousal classification relies more on brain regions associated with visual stimulus processing. In contrast, valence classification involves more excellent processing of social–emotional information and internal emotional regulation. Furthermore, activity in motor-related brain regions indicates a significant role of bodily expression in valence regulation. These findings provide vital insights for the further exploration of the application of single-channel EEG signals in emotion recognition, particularly regarding how specific emotional dimensions can be captured through various channels. Future research should explore the associations of such channels with other potential functional areas and their collaborative roles in the complex process of individual emotion recognition.

### 4.4. Comparative Study

To thoroughly investigate the benefits of the proposed method, a comparative study with recent works is performed, as outlined in Table 5. As seen, although the classification accuracies of the proposed method are somewhat lower than those of methods using multiple channels and complex deep learning networks, such as a fuzzy rank-based deep learning model [56], CNN [10,57], Multi-Layer Perceptron (MLP) [57], and LSTM [58,59], managing to deliver remarkable performance through single-channel data inputs, reducing dimensionality and computational cost. On the one hand, the CNN-based deep learning approach [57] achieves classification accuracies of 94.33% for arousal and 93.53% for valence, highlighting the potential of multi-channel configurations and deep learning networks in sophisticated emotion recognition. Other model-based networks like MLP [57] also display high accuracy, offering 94.25% for arousal and 93.39% for valence. On the other hand, conventional approaches, such as SVM [60], logistic regression [61], and Minimum Distance to Riemannian Mean (MDRM) [62], present comparatively lower performance. For instance, MDRM combined with Principal Component Analysis (PCA) [62] achieves only 57.42% for arousal and 64.06% for valence with 60 s data. Such disparities indicate the vital roles of feature selection and fusion in EEG-based emotion recognition. In this regard, the proposed method established by the BREM derived from multi-entropy fusion and similarity measures has been successfully implemented to recognize various emotions through a single-channel approach, emphasizing a balance between channel number and classification accuracy. Hence, such performances are more outstanding than those reported in previous studies, considering fewer data resources as a concern, facilitating the resource-efficient design of low-cost portable emotion-aware devices.

### 4.5. Discussion

First, the proposed method is validated and analyzed using the publicly available DEAP dataset in this work. The findings indicate that the classification accuracy gradually increases as the time length decreases. This trend is particularly evident when utilizing 5 s time segments, during which classification performance peaks. Such an observation suggests that shorter time segments help assess transient emotional responses. Longer time segments, despite containing more EEG signals, may include disturbances unrelated to the emotion, which could reduce the accuracy. The results from shorter time segments also exhibit lower standard deviations, revealing more stable outcomes for emotion recognition.

Second, when comparing various methods for measuring similarity levels between the BREMs derived from multi-entropy fusion, it becomes apparent that DTW is appropriate for this context, outperforming other similarity measures through its refined analytical capabilities. DTW excels in capturing nonlinear temporal variations, which are beneficial for identifying subtle emotional changes over time through the BREMs. Unlike SCC and JSC, which assume a more straightforward relationship between the signals, DTW can account for time shifts and distortions, making it highly effective for processing non-stationary biomedical signals such as EEG, where emotional states usually evolve dynamically.

Third, the investigation demonstrates that the inputs from individual channels achieve higher classification accuracy than those from the same region, reaffirming that individual channels can more directly capture EEG signals pertinent to emotions. Meanwhile, the appropriate time segments and channels for arousal and valence vary among subjects, reflecting the functional diversity of emotional dimensions across different brain parts. Thus, the proposed method provides valuable insights for choosing suitable time lengths and channels in EEG-based emotion recognition, laying the groundwork for future resource-efficient hardware designs.

Finally, this work exhibits several limitations despite demonstrating the advantage of multi-entropy fusion. The complexity and variability in emotional responses indicate that individual differences are significant. While several subjects exhibit better classification results with short time segments, others achieve better results with longer segments, such as 10 s. Future work could explore the association between individual differences and time lengths, particularly the potential links between emotional dimensions and individual neural responses regarding EEG-based entropy fusion views.

## 5. Conclusions

This work proposes an innovative resource-efficient multi-entropy fusion method for EEG-based emotion recognition in which the development of the BREM involves the extraction of five brain rhythms through DWT, followed by acquisition of multi-entropy features that encapsulate the inherent complexity and dynamics of EEG signals. Subsequently, DTW emerges as a suitable method for measuring similarities among BREM samples. The focused evaluation of time window analysis reveals that the 5 s EEG segment yields robust data for recognition. Although most methods select 32-channel or other multi-channel setups as input to capture abundant brain information to enhance emotion recognition performance, the proposed method using the single-channel mode provides a promising, resource-efficient way to simplify the EEG setup without compromising classification performance. The experimental results from the DEAP dataset demonstrate that single-channel data achieve 84.62% and 82.48% accuracy in arousal and valence classification tasks, respectively, underscoring the effectiveness of BREMs from multi-entropy fusion. Therefore, such performances are meaningful when considering fewer data resources as a concern, which opens the possibility of an entropy fusion method that can helps to design portable EEG-based emotion-aware devices for daily usage.

While the results are particularly interesting, further refinement and validation of the proposed method across diverse scenarios are desirable, especially with a single-channel EEG setup, which may not fully capture the complexity of emotional responses across different brain regions. Therefore, future work should explore ways to combine the resource-efficiency of single-channel EEG with the comprehensive spatial coverage provided by multi-channel EEG, improving performance for more nuanced emotional analysis. In addition, it is vital to focus on exploring real-time classification by integrating multimodal data and customizing the methodology to accommodate individual differences in emotional processing and validating its robustness using datasets beyond DEAP. Such efforts will assess the method’s performance across diverse experimental conditions, enhancing its applicability in broader real-world scenarios.

## Figures and Tables

**Table 1:** Key information of the DEAP dataset.

Number of Subjects	32
Age	27.19 ± 4.45 years
Number of subjects	15 females + 17 males
Number of experimental trials per subject	40
Experimental stimuli	Music videos from YouTube
Time duration per trial	One minute
Number of EEG channels	32
Sampling frequency (Hz)	128

**Table 2:** Specific EEG channels in each brain region.

Brain Region	EEG Channels
Frontal	FP1, FP2, AF3, AF4, F3, FZ, F4
Central	FC5, FC1, FC2, FC6, C3, CZ, C4
Parietal	CP5, CP1, CP2, CP6, P3, PZ, P4
Temporal	F7, F8, T7, T8, P7, P8
Occipital	PO3, PO4, O1, OZ, O2

**Table 3:** The classification results (mean ± standard deviation (%)) using different time windows, similarity measures, and data input modes.

Similarity Measure Method	Time Window (s)	Classification Accuracy (Mean ± Standard Deviation (%))			
		Single-Channel Mode		Single-Region Mode	
		Arousal	Valence	Arousal	Valence
DTW	30	80.92 ± 4.38	78.95 ± 3.27	78.29 ± 5.43	76.23 ± 3.81
	20	82.24 ± 4.28	79.85 ± 3.18	80.51 ± 5.08	78.54 ± 4.68
	10	83.06 ± 4.77	81.42 ± 3.40	80.92 ± 4.82	78.29 ± 3.41
	5	84.62 ± 4.39	**82.48 ± 2.88**	**83.06 ± 5.13**	**79.69 ± 2.93**
MI	30	81.25 ± 5.62	78.78 ± 3.89	79.36 ± 6.27	77.06 ± 3.68
	20	83.31 ± 4.80	79.36 ± 3.21	79.61 ± 5.71	76.97 ± 3.34
	10	83.72 ± 4.25	80.51 ± 2.40	81.42 ± 4.86	78.62 ± 3.39
	5	**84.79 ± 5.08**	81.99 ± 3.07	82.24 ± 4.87	79.61 ± 3.13
SCC	30	66.37 ± 12.45	62.34 ± 9.56	70.56 ± 13.17	68.42 ± 8.21
	20	65.87 ± 13.31	62.17 ± 8.71	70.64 ± 12.81	67.93 ± 8.06
	10	65.46 ± 14.38	62.58 ± 9.16	70.39 ± 13.33	68.09 ± 8.71
	5	68.75 ± 13.97	65.54 ± 7.33	71.96 ± 11.88	68.91 ± 8.41
JSC	30	59.38 ± 16.26	56.91 ± 9.70	59.38 ± 16.26	56.91 ± 9.70
	20	59.38 ± 16.26	56.91 ± 9.70	59.38 ± 16.26	56.91 ± 9.70
	10	59.38 ± 16.26	56.91 ± 9.70	59.38 ± 16.26	56.91 ± 9.70
	5	59.38 ± 16.26	56.91 ± 9.70	59.38 ± 16.26	56.91 ± 9.70

**Table 4:** The appropriate time segments and channels of 32 subjects from the DEAP dataset for arousal and valence classifications.

Subject	Arousal			Valence		
	Channel	Time Segment (s)	Classification Accuracy (%)	Channel	Time Segment (s)	Classification Accuracy (%)
S1	T8	15–20	84.21	CP5	5–10	84.21
S2	FC5	30–35	81.58	O2	5–10	81.58
S3	PO4	10–15	89.47	FC2	20–25	84.21
S4	CP6	0–5	84.21	AF4	30–35	84.21
S5	PO3	5–10	81.58	P3	35–40	81.58
S6	CZ	20–25	81.58	F4	20–25	89.47
S7	PO3	15–20	84.21	FC6	5–10	86.84
S8	F4	15–20	81.58	P7	10–15	81.58
S9	CP1	5–10	78.95	F7	30–35	81.58
S10	F8	0–5	78.95	FC6	25–30	81.58
S11	P4	25–30	81.58	F3	5–10	81.58
S12	FC1	20–25	92.11	P3	10–15	78.95
S13	F4	0–5	92.11	PO3	0–5	81.58
S14	F7	5–10	86.84	AF3	0–5	81.58
S15	CP5	5–10	78.95	FC5	25–30	81.58
S16	P8	30–35	84.21	FC2	0–5	81.58
S17	C4	45–50	81.58	F8	50–55	81.58
S18	CP1	40–45	84.21	C4	0–5	81.58
S19	F3	20–25	84.21	T8	25–30	81.58
S20	P8	35–40	89.47	C3	10–15	81.58
S21	PO3	0–5	94.74	FZ	20–25	78.95
S22	FP1	15–20	84.21	CP1	40–45	81.58
S23	FZ	35–40	86.84	C3	30–35	89.47
S24	O2	25–30	92.11	PZ	35–40	81.58
S25	PO4	50–55	86.84	CP5	0–5	78.95
S26	FP1	50–55	84.21	F7	10–15	81.58
S27	CP1	0–5	81.58	F7	5–10	86.84
S28	F7	5–10	78.95	F4	20–25	81.58
S29	AF4	35–40	86.84	FC1	15–20	81.58
S30	PO3	35–40	78.95	FZ	50–55	86.84
S31	FC2	20–25	81.58	C4	20–25	84.21
S32	O2	0–5	89.47	F3	20–25	76.32

**Table 5:** Comparison with recent works.

	Time Window (s)	Number of Channel	Main Methodology	Classification Accuracy (%)	
				Arousal	Valence
Akhand et al. [10]	8	32	Connectivity feature map with CNN	91.66	91.29
Dhara et al. [56]	2	14	Fuzzy rank-based deep learning approach using Gompertz function	91.65	90.84
Kumar and Molinas [57]	1	32	Differential entropy with MLP	94.25	93.39
			Differential entropy with CNN	94.33	93.53
Gaddanakeri et al. [58]	60	14	Brain rhythms with LSTM (S1–S22)	82.40	78.28
			Brain rhythms with LSTM (S23–S32)	63.15	62.06
Singh et al. [59]	3	5	Grey Wolf Optimization (GWO) and LSTM with data augmentation	81.25	92.50
Jha et al. [60]	60	32	Brain rhythms with SVM	70.88	76.00
Pan et al. [61]	1	32	Logistic regression with Gaussian kernel and Laplacian prior	77.03	77.17
Al-Mashhadani et al. [62]	60	32	MDRM-PCA	57.42	64.06
This work	5	1	BREM from multi-entropy fusion and similarity measure by DTW	84.62	82.48

## Data Availability

The datasets generated and/or analyzed during the current study are available at https://github.com/zyzc75/BREM-SIMILARITY.git (accessed on 18 January 2025).
